# Association between Plasma Neutrophil Gelatinase Associated Lipocalin Level and Obstructive Sleep Apnea or Nocturnal Intermittent Hypoxia

**DOI:** 10.1371/journal.pone.0054184

**Published:** 2013-01-14

**Authors:** Kimihiko Murase, Kiyoshi Mori, Chikara Yoshimura, Kensaku Aihara, Yuichi Chihara, Masanori Azuma, Yuka Harada, Yoshiro Toyama, Kiminobu Tanizawa, Tomohiro Handa, Takefumi Hitomi, Toru Oga, Michiaki Mishima, Kazuo Chin

**Affiliations:** 1 Department of Respiratory Medicine, Graduate School of Medicine, Kyoto University, Kyoto, Japan; 2 Department of Medicine and Clinical Science, Graduate School of Medicine, Kyoto University, Kyoto, Japan; 3 Department of Respiratory Care and Sleep Control Medicine, Graduate School of Medicine, Kyoto University, Kyoto, Japan; 4 Department of Respiratory Medicine, Red Cross Otsu Hospital, Shiga, Japan; I2MC INSERM UMR U1048, France

## Abstract

**Background:**

Both obstructive sleep apnea (OSA) and a novel lipocalin, neutrophil gelatinase associated lipocalin (Ngal), have been reported to be closely linked with cardiovascular disease and loss of kidney function through chronic inflammation. However, the relationship between OSA and Ngal has never been investigated.

**Objectives:**

To evaluate the relationship between Ngal and OSA in clinical practice.

**Methods:**

In 102 patients, polysomnography was performed to diagnose OSA and plasma Ngal levels were measured. The correlations between Ngal levels and OSA severity and other clinical variables were evaluated. Of the 46 patients who began treatment with continuous positive airway pressure (CPAP), Ngal levels were reevaluated after three months of treatment in 25 patients.

**Results:**

The Ngal level correlated significantly with OSA severity as determined by the apnea hypopnea index (r = 0.24, p = 0.01) and 4% oxygen desaturation index (ODI) (r = 0.26, p = 0.01). Multiple regression analysis showed that the Ngal level was associated with 4%ODI independently of other clinical variables. Compliance was good in 13 of the 25 patients who used CPAP. Although the OSA (4%ODI: 33.1±16.7 to 1.1±1.9/h, p<0.01) had significantly improved in those with good compliance, the Ngal levels were not significantly changed (60.5±18.1 before CPAP vs 64.2±13.9 ng/ml after CPAP, p = 0.27).

**Conclusions:**

Plasma Ngal levels were positively associated with the severity of OSA. However, the contribution rate of OSA to systemic Ngal secretion was small and changes in Ngal levels appeared to be influenced largely by other confounding factors. Therefore, it does not seem reasonable to use the Ngal level as a specific biomarker of OSA in clinical practice.

## Introduction

Neutrophil gelatinase associated lipocalin (Ngal), also known as lipocalin 2, is a 25-kDa secretory glycoprotein that was originally identified in human neutrophils. This protein was originally known as an innate immunity antibacterial factor released by activated neutrophils. [1], [2] It has also become known to be produced by renal tubular cells in response to different types of injury. [3] Based on experimental and clinical findings, Ngal is widely considered as an excellent indicator of acute and chronic kidney injury.[3]–[7] Moreover, because this protein is also released by endothelial cells and failing myocardium, a close relationship between blood Ngal levels and heart failure or cardiovascular diseases has been suggested.[8]–[10].

Obstructive sleep apnea (OSA) is a highly prevalent disorder, affecting about 4–20% of adults and is characterized by repetitive episodes of partial or complete obstruction of the upper airway during sleep associated with transient oxygen desaturation.[11]–[13] Accumulating clinical evidence suggests that OSA is an independent risk factor for cardiovascular disease and loss of kidney function through nocturnal hypoxia and chronic inflammation.[14]–[17] From an in vitro model of OSA, it was suggested that the pro-inflammatory transcription factor, nuclear factor-kappa B (NF-κB), plays an important role in the inflammatory process of a cell’s reaction to intermittent hypoxia/reoxygenation. [18] Meanwhile, it has been reported that several inflammatory stimuli, such as interleukin 1β, stimulate systemic Ngal expression and secretion. NF-κB also has been shown to transactivate Ngal expression, suggesting that Ngal might be involved in inflammatory responses. [19], [20].

Therefore, a positive correlation between OSA severity and systemic Ngal secretion through chronic inflammation seems possible. However, this relationship has never been investigated. Thus, we hypothesized that blood Ngal levels are elevated in patients with OSA and that its levels are modified by the treatment of OSA with continuous positive airway pressure (CPAP). In the present study, we measured plasma Ngal levels in patients with OSA and evaluated its utility in clinical practice.

## Methods

### Subjects

Study patients were consecutively recruited from the Sleep Unit of Kyoto University Hospital between January 2009 and May 2012. All had been referred to our sleep unit under suspicion of OSA with symptoms such as habitual snoring or daytime sleepiness. None had been previously diagnosed with or treated for OSA. Patients with overt renal failure (serum creatinine >1.3 mg/dl) or with any history of cardiovascular diseases, heart failure or arrhythmia were excluded because severe renal and/or heart failure can directly affect plasma Ngal levels. Also excluded were patients with pulmonary diseases, chronic infection, history of cancer or collagen disease. Since a consensus about the relationship between Ngal levels and metabolic syndrome has not yet been formed, we aimed to evaluate the correlations between risk factors for metabolic syndrome and plasma Ngal levels in actual clinical practice. We did not exclude patients with components of metabolic syndrome such as hypertension, diabetes and dyslipidemia even if they were under treatment for these comorbidities.[21]–[23] This study was approved by Kyoto University Graduate School and Faculty of Medicine Ethics Committee, and written informed consent was obtained from all patients.

### Polysomnography and CPAP Implementation

The diagnosis of OSA was confirmed by polysomnography (SomnoStar pro, Cardinal Health, Dublin, OH, USA or Alice 4, Philips Respironics, Inc., Murrysville, PA, USA), which was started at 22:00 and ended at 6:00 the following morning. Surface electrodes were attached using standard techniques to obtain an electrooculogram, electromyogram of the chin and 12-lead electroencephalograph. Sleep stages were defined according to the criteria of Rechtchaffen and Kales. [24] Ventilation was monitored by inductive plethysmography (Respitrace QDC, Viasys Healthcare, Palm Springs, CA, USA). Airflow was monitored by a nasal pressure transducer and supplemented by an oronasal thermal sensor. Arterial oxygen saturation (SpO_2_) was monitored continuously with a pulse oximeter.

Apnea was defined as the continuous cessation of airflow for more than 10 seconds and hypopnea was defined as a reduction in airflow of 30% or more lasting for 10 seconds or more accompanied by a decrease in SpO_2_ of at least 4%. [25] Apnea-hypopnea index (AHI) values were calculated as the number of episodes of apnea and hypopnea per hour over the total sleep time. 4% oxygen desaturation index (ODI) values were defined as the number of desaturations ≥4% per hour of sleep. The length of time SpO_2_<90% during sleep was calculated in each patient. Patients with central sleep apnea were excluded. OSA severity was defined by the AHI as follows: non OSA (AHI<5), mild OSA (5≤AHI<15), moderate OSA (15≤AHI<30) and severe OSA (30≤AHI).

Patients with an AHI ≥15 were candidates for nasal CPAP. Those who agreed with CPAP implementation underwent a second polysomnography with CPAP titration. We implemented CPAP with the auto adjusting positive airway pressure (PAP) function for all patients. Based on the second sleep study, minimum and maximum PAP were determined to abolish all respiratory events, arousal and desaturation events.

### Follow-Up

At the three-month follow-up, we urged the patients to undergo a third sleep study to confirm whether an adjustment of the CPAP setting was necessary. To investigate the effect of CPAP treatment on plasma Ngal levels, at the third sleep study blood samples were collected in the same way as at the first sleep study. We also checked use time of the CPAP machine by reading the time counter on the CPAP machines. Similar to prior studies, we defined ‘good compliance’ as the use of CPAP for >4 h per night on >70% of nights and categorized the patients into two groups, those with ‘good compliance’ or ‘poor compliance’. [26] We analyzed the data separately for each group and compared clinical variables before and after CPAP treatment.

### Blood Sampling and Measurement of Plasma Ngal Level

Blood samples were drawn at 7:00 in the morning after the subjects had fasted beginning at 20:00 the previous night. Blood samples were centrifuged immediately at 3,000 rpm at 4°C for 10 min. The separated samples were stored −80°C until assay. Plasma Ngal concentrations were determined by an ELISA kit provided by Bioporto Diagnostics, Gentofte, Denmark. Intra- and inter-assay coefficients of variation for Ngal were 1.2–4.0% and 2.2–11.2%, respectively.

### Definition of Metabolic Syndrome

In classifying patients based on the components of metabolic syndrome, we utilized Japanese criteria. [27] Waist circumference (WC) was measured at the level of the navel with the patient standing, and visceral fat accumulation was determined to be positive at WC ≥85 cm for men and ≥90 cm for women. A diagnosis of metabolic syndrome required the subject to have visceral fat accumulation and 2 or 3 of the following: (a) dyslipidemia (triglycerides ≥150 mg/dL and/or high-density lipoprotein cholesterol level <40 mg/dL, or specific treatment for these lipid abnormalities); (b) hypertension (systolic blood pressure ≥130 mmHg and/or diastolic blood pressure ≥85 mmHg, or treatment of previously diagnosed hypertension); and (c) hyperglycemia (fasting plasma glucose ≥110 mg/dL or specific treatment for diabetes mellitus). Anthropometric parameters and blood pressure were measured immediately after polysomnography recording ended.

### Statistical Analysis

In the analysis of data, we classified the patients depending on the severity of OSA and compared their clinical backgrounds. We also compared plasma Ngal levels between patients with and without each component of metabolic syndrome to investigate the relationships between plasma Ngal levels and metabolic syndrome. Data were expressed as means ± standard deviation. The significance of intergroup differences based on the severity of OSA was determined by an analysis of variance. When a significant difference was found, we used the Tukey’s honestly significant difference procedure to identify where the difference was significant. A chi-square test and the Mann-Whitney U test were used to compare categorical and continuous variables, respectively. We used Pearson’s coefficient tests to evaluate the relationship between the plasma Ngal level and other continuous variables. Based on the results of this analysis, multiple regression analyses were performed to clarify the contribution rate of OSA and other comorbidities to systemic Ngal secretion. Wilcoxon signed rank test was used to compare clinical variables before and after CPAP treatment. Two-tailed p-values <0.05 were considered statistically significant. All statistical analyses were performed using JMP 7.0.2 statistical software (SAS Institute Inc., Cary, NC, USA).

## Results

### Baseline Characteristics of Study Patients

A total of 102 patients were studied, and their baseline characteristics are shown in table 1.Those with severe OSA were characterized by a significantly higher body mass index (BMI) than in the other three groups. The percentages of patients who fulfilled the criterion for visceral fat accumulation increased as the severity of OSA increased. Other anthropometric parameters with significant differences among the groups are also shown in table 1. With the exception of the parameters for OSA, there were no significant differences among the four groups in other clinical background factors. (Tables 1 and 2).

**Table 1:** Baseline characteristics and data on metabolic syndrome and its components in study patients.

	non OSA (n = 15)	mild OSA (n = 37)	moderate OSA (n = 24)	severe OSA (n = 26)	p
Age (y)	48.2±17.2	55.1±13.3	57.3±14.5	58.6±11.5	0.12
Sex (male), n(%)	8 (53.3)	24 (64.9)	18 (75)	18 (69.2)	0.56
Smoking status never/ex/current, n	2/3/10	5/13/19	3/5/16	3/8/15	0.88
Body mass index (kg/m^2^)	23.5±3.7	25.1±4.6	24.8±4.1	29.2±7.8a,b,c	<0.01
Neck circumference (cm)	36.2±3.1	37.9±3.7	37.8±3.6	39.8±4.0^a^	0.03
Waist circumference (cm)	84.3±11.5	90.1±13.1	89.7±11.0	98.6±14.4a,b	0.01
Hip circumference (cm)	90.3±8.8	94.6±10.6	92.1±9.3	101.3±15.3a,c	0.01
Waist-to-hip ratio	0.93±0.07	0.95±0.05	0.97±0.04	0.97±0.03	0.04
SBP (mmHg)	119.8±16.1	124.2±16.3	127.3±12.9	127.4±16.0	0.40
DBP (mmHg)	72.7±11.9	76.7±11.5	78.6±11.2	76.2±12.0	0.50
**Percentages of patients with metabolic syndrome or components of metabolic syndrome**					
Hypertension, n (%)	7 (46.7)	20 (54.1)	15 (62.5)	19 (73.1)	0.16
Hyperglycemia, n (%)	5 (33.3)	7 (18.9)	4 (16.7)	5 (19.2)	0.56
Dyslipidemia, n(%)	6 (40.0)	16 (43.2)	11 (45.8)	13 (50.0)	0.98
Visceral fat accumulation, n(%)	7 (46.7)	22 (59.5)	18 (75.0)	23 (88.5)	0.01
Metabolic syndrome, n(%)	5 (33.3)	13 (35.1)	8 (33.3)	11 (42.3)	0.90
**Percentages of patients under treatment for components of metabolic syndrome**					
Hypertension, n (%)	2 (13.3)	14 (37.8)	9 (37.5)	12 (46.2)	0.16
Diabetes, n (%)	2 (13.3)	3 (8.1)	2 (8.3)	5 (19.2)	0.56
Dyslipidemia, n(%)	4 (26.7)	8 (21.6)	5 (20.8)	6 (23.1)	0.98

Data are expressed in mean ± SD or n (%).

OSA: obstructive sleep apnea; SBP: systolic blood pressure; DBP: diastolic blood pressure;

a p<0.05 vs non OSA; ^b^p<0.05 vs mild OSA; ^c^p<0.05 vs moderate OSA.

**Table 2:** OSA parameters and laboratory profiles.

	non OSA (n = 15)	mild OSA (n = 37)	moderate OSA (n = 24)	severe OSA (n = 26)	p
**Parameters of OSA**					
Apnea hypopnea index/h	2.2±1.6	9.4±2.6	22.2±5.0a,b	50.5±19.8a,b,c	<0.01
4%ODI/h	1.8±1.6	8.2±3.3	20.7±6.0a,b	49.6±20.2a,b,c	<0.01
Minimum SpO_2_ (%)	90.3±4.5	84.9±4.3	78.4±8.6a,b	71.3±12.2a,b,c	<0.01
Arousal index/h	24.3±12.1	22.8±9.6	30.7±13.2	43.4±18.3a,b,c	<0.01
Length of time SpO_2_<90% (m)	3.2±5.4	11.4±17.4	37.3±52.2	121.1±107.8a,b,c	<0.01
**Laboratory profiles**					
FPG (mg/dl)	107.7±39.9	95.4±20.1	96.4±24.3	102.5±20.2	0.35
HbA1c (%)	5.80±1.22	5.49±0.65	5.45±0.91	5.74±0.86	0.45
Total cholesterol (mg/dl)	185.5±32.3	195.2±35.4	203.0±44.0	201.5±44.0	0.53
LDL cholesterol (mg/dl)	103.2±27.9	116.2±27.8	118.5±36.8	109.5±31.8	0.40
HDL cholesterol (mg/dl)	55.2±12.9	52.8±13.5	53.0±16.1	51.9±13.9	0.91
Triglycerides (mg/dl)	125.1±86.5	119.8±58.0	139.1±79.1	168.0±185.2	0.39
BNP (pg/ml)	14.4±8.7	20.6±24.4	22.4±31.8	21.5±18.7	0.73
Creatinine (mg/dl)	0.72±0.19	0.74±0.15	0.80±0.17	0.78±0.21	0.45
Ngal (ng/ml)	46.9±6.0	48.9±10.9	51.3±15.2	55.4±16.7	0.16

Data are expressed in mean ± SD or n (%).

OSA: obstructive sleep apnea; ODI: oxygen desaturation index; SpO_2_: saturation of oxygen; FPG: fasting plasma glucose; LDL: low density lipoprotein; HDL: high density lipoprotein; BNP: brain natriuretic peptide; Ngal: neutrophil gelatinase associated lipocalin.

a p<0.05 vs non OSA; ^b^p<0.05 vs mild OSA; ^c^p<0.05 vs moderate OSA.

### Plasma Ngal Levels in Patients at Diagnosis and follow up

**Figure 1:**
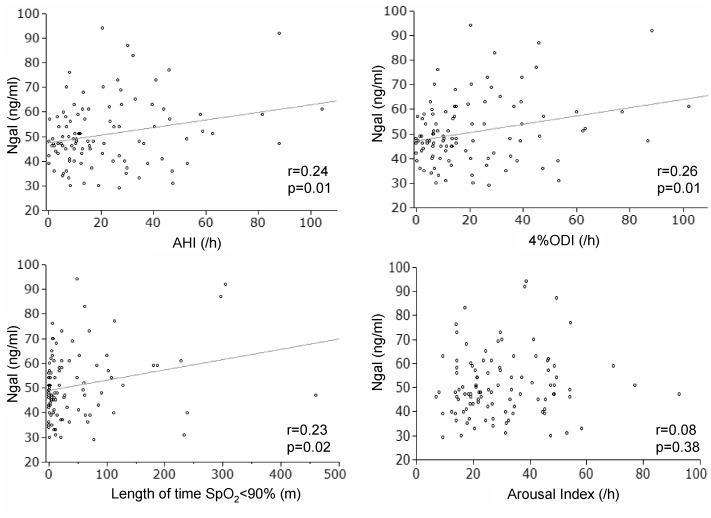
Simple correlations between plasma neutrophil gelatinase associated lipocalin (Ngal) levels and parameters of obstructive sleep apnea. AHI: apnea hypopnea index; ODI: oxygen desaturation index; SpO_2_: saturation of oxygen.

Table 2 shows baseline plasma Ngal levels in the four groups, with no statistically significant differences found among them. However, simple linear regression analysis showed significant correlations of the plasma Ngal level with the following parameters of OSA: AHI (r = 0.24, p = 0.01), 4%ODI (r = 0.26, p = 0.01) and time of SpO_2_<90% (r = 0.23, p = 0.02). (Figure 1) The plasma Ngal level was also correlated with values for serum low density lipoprotein (LDL) cholesterol (r = −0.31, p<0.01), triglycerides (r = 0.24, p = 0.01) and creatinine (r = 0.34, p<0.01). On the other hand, none of anthropometric parameters and parameters associated with diabetes such as fasting plasma glucose and HbA1c levels showed significant correlations with Ngal levels.(Table 3) Furthermore, in the present cohort, significant differences were not found in plasma Ngal levels between patients with and without each of the components of metabolic syndrome. (Table 4).

**Table 3:** Simple correlations between plasma neutrophil gelatinase associated (Ngal) levels and clinical variables.

	r	p
Age (y)	0.04	0.62
Body mass index (kg/m^2^)	0.13	0.17
Neck circumference (cm)	0.00	0.84
Waist circumference (cm)	0.00	0.91
Hip circumference (cm)	0.00	0.99
Waist-to-hip ratio	0.03	0.76
SBP (mmHg)	0.00	0.96
DBP (mmHg)	−0.13	0.17
FPG(mg/dl)	−0.08	0.39
HbA1c (%)	−0.06	0.52
Total cholesterol (mg/dl)	0.12	0.21
LDL-cholesterol (mg/dl)	−0.31	<0.01
HDL-cholesterol (mg/dl)	−0.18	0.06
Triglycerides (mg/dl)	0.24	0.01
BNP (pg/ml)	0.00	0.74
Creatinine (mg/dl)	0.34	<0.01

r: correlation coefficient; SBP: systolic blood pressure; DBP: diastolic blood pressure; FPG: fasting plasma glucose; LDL: low density lipoprotein; HDL: high density lipoprotein; BNP: brain natriuretic peptide.

**Table 4:** Plasma Ngal levels in patients with and without each component of metabolic syndrome.

	Plasma Ngal levels (ng/ml)		
	Comorbidity(+)	Comorbidity(−)	p
Hypertension	52.7±1.7	48.0±2.1	0.17
	(n = 61)	(n = 41)	
Hyperglycemia	48.4±14.5	51.4±13.0	0.27
	(n = 21)	(n = 81)	
Dyslipidemia	51.9±14.7	49.9±12.2	0.49
	(n = 46)	(n = 56)	
Visceral fat accumulation	50.9±14.1	50.5±11.6	0.92
	(n = 70)	(n = 32)	
Metabolic syndrome	51.8±16.7	50.2±11.1	0.93
	(n = 37)	(n = 65)	

Data are expressed in mean ± SD.

Ngal: neutrophil gelatinase associated lipocalin.

For the multiple regression analysis, we chose 4%ODI as the representative variable for OSA severity as it had the best correlation with the Ngal level among OSA parameters in the simple correlation analysis. The analysis demonstrated that 4%ODI was associated with the Ngal level independently of creatinine and LDL-cholesterol levels. The contribution rate of 4% ODI to the Ngal level was 6.2% (Table 5).

**Table 5:** Multiple regression analyses using plasma neutrophil gelatinase associated lipocalin (Ngal) level as a dependent variable.

	p	β	r	R^2^(%)
Body mass index (kg/m^2^)	0.39	–		
4%ODI/h	<0.01	0.24	0.26	6.2
LDL-cholesterol (mg/dl)	<0.01	−0.29	−0.31	9.0
HDL-cholesterol (mg/dl)	0.71	–		
Triglycerides (mg/dl)	0.31	–		
Creatinine (mg/dl)	<0.01	0.28	0.34	9.5
Cumulative R^2^				24.7

β: standard regression coefficient; r: correlation efficient; R^2^: contribution rate; ODI: oxygen desaturation index; LDL: low density lipoprotein; HDL: high density lipoprotein.

CPAP was implemented for 46 of the 50 patients with moderate or severe OSA. Of the 46 patients, 27 agreed to a follow-up sleep study. Just before the reevaluation, cardiac medicine was prescribed for one patient and an upper airway infection was found in another patient. These two patients were excluded from the analysis, and the remaining 25 patients were reevaluated. Thirteen were categorized into the good compliance group and the other 12 patients into the poor compliance group. Those in the good compliance group were significantly older than patients in the poor compliance group. The determined maximum and minimum PAP did not differ between the two groups.

After CPAP implementation, OSA was significantly improved in both groups. In the good compliance group, despite improvements in OSA, no significant change was noted in plasma Ngal levels from values before CPAP use. Furthermore, in the poor compliance group, Ngal levels were significantly elevated after CPAP implementation. There were not significant differences in the other confounding factors before and after CPAP treatment (Table 6).

**Table 6:** Changes in clinical variables from baseline to after CPAP implementation.

	CPAP good compliance (n = 13)			CPAP poor compliance (n = 12)			
	before CPAP	after CPAP	p*	before CPAP	after CPAP	p*	p^#^
Ngal (ng/ml)	60.5±18.1	64.2±13.9	0.27	52.8±16.8	63.1±14.2	<0.01	–
4%ODI (/h)	33.1±16.7	1.1±1.9	<0.01	41.5±22.5	1.5±2.3	<0.01	–
Creatinine (mg/dl)	0.85±0.20	0.88±0.19	0.13	0.77±0.21	0.79±0.20	0.25	–
LDL cholesterol (mg/dL)	107.5±32.7	103.8±30.8	0.70	115.2±28.2	121.0±28.0	0.58	–
Body mass index (kg/m^2^)	23.9±2.0	23.9±2.2	0.85	28.2±6.6	28.6±6.6	0.14	
Age (y)	67.5±8.3		–	54.5±12.2		–	0.01
Days with CPAP use >4 h (%)	85.8±9.6		–	43.3±20.7		–	<0.01
Maximum PAP (cmH_2_O)	9.9±2.8		–	10.8±2.5		–	0.62
Minimum PAP (cmH_2_O)	4.5±0.9		–	4.7±0.8		–	0.35

Data are expressed in mean±SD.

CPAP: continuous positive airway pressure; Ngal: neutrophil gelatinase associated lipocalin; ODI: oxygen desaturation index; LDL: low density lipoprotein; PAP: positive airway pressure; p*: p value for comparison with values before and after CPAP treatment; p^#^ : p value for comparison between CPAP good compliance and CPAP poor compliance groups.

## Discussion

In this cross sectional evaluation, although significant differences in plasma Ngal levels were not found among groups classified according to the severity of OSA, parameters of OSA, such as 4%ODI and AHI per se, correlated with plasma Ngal levels in regression analysis. This suggests that OSA contributes, although weakly, to elevated plasma Ngal levels through nocturnal hypoxia. Because it has been reported that hypoxia induces an elevation in plasma Ngal levels in an experimental animal model, it is possible that OSA induces Ngal elevation through nocturnal intermittent hypoxia. [28] To the best of our knowledge, this is the first report to evaluate the relationship between the Ngal protein level and OSA severity in clinical practice.

The relationship between another protein in the lipocalin family and OSA has been investigated. Makino et al and Nena et al, respectively, investigated the relationship between the plasma level of retinol binding protein 4 (RBP-4), which also belongs to the lipocalin protein family, and OSA. [29], [30] However, neither study found a correlation between RBP-4 levels and apnea-related indices. Although both Ngal and RBP-4 belong to the lipocalin family and share a common tertiary structure, these two proteins appear to have different patterns of regulation in response to inflammatory mediators. [21], [23].

Our results also demonstrated a significant inverse correlation between Ngal and LDL cholesterol levels. Wallenius et al also reported such an inverse correlation in their epidemiological study. [21] However, in other studies, correlations between these two variables were not found. [23], [31], [32] Although this inverse correlation is possible, the results seem to vary depending on the clinical characteristics of the examined cohorts. Furthermore, the mechanisms of this correlation remain utterly unknown.

The relationship between Ngal and metabolic syndrome is quite controversial. Whereas Wang et al and Yan et al reported a close association between Ngal and obesity or insulin resistance, Wallenius et al found no correlation between these risk factors. [21], [22], [31] Our results did not show any significant correlation between Ngal levels and obesity or diabetic indices. Also, in the present cohort there were no significant differences in Ngal levels between patients with and without metabolic syndrome. These results also seem to depend on the clinical characteristics of the cohorts. Specifically, we did not exclude patients under treatment for metabolic syndrome to investigate the utility of the plasma Ngal level in actual clinical practice. Because it was reported that the pharmaceutical treatment of diabetes and dyslipidemia can change plasma Ngal levels, treatment of metabolic syndrome in our cohort possibly affected the results. [31], [33] Furthermore, in most of these studies, renal function was not taken into account as an explanatory variable of the Ngal level. Risk factors for metabolic syndrome induce latent renal function impairment and even a subtle change in renal function is known to affect blood and urinary Ngal levels. [34] Giaginis et al reported that plasma Ngal levels were higher in patients with than without hypertension and they speculated that the association between elevated Ngal levels and hypertension is secondary to the confounding effect of renal impairment. [35] In our study, even though we did not include patients with overt renal failure, the plasma Ngal levels correlated significantly with serum creatinine levels. Therefore, in evaluating the direct link between metabolic syndrome and Ngal levels, statistical correction for renal function seem to be necessary. In fact, Liu et al reported that the significant correlation between Ngal and insulin resistance detected in their study cohort disappeared after adjustment for serum creatinine values. [32].

Contrary to our expectation, plasma Ngal levels did not change even with the appropriate use of CPAP that effected an improvement in OSA. We could not confirm a direct causality between Ngal levels and OSA. Although we based the three-month treatment observation period on experiences in previous studies, we might need an extended period to find more remarkable changes in Ngal levels in the present cohort. [23], [31].

Ngal levels were elevated after CPAP implementation in patients with poor compliance with CPAP. Because we did not include an actual control group that did not use CPAP, we could not judge whether the change in Ngal levels was caused by incomplete CPAP use or other reasons. Although we took every conceivable confounding factor into account, other determinants that we were not aware of might have influenced the results. In the cross sectional studies, the contribution rate of 4%ODI to Ngal levels was not large (6.2%). Therefore, it seems quite possible that other determinants negated the influence of the improvement in OSA. The presence of certain components of metabolic syndrome and their treatment in the present cohort might be among these determinants.

We recognize several limitations in the present study. First, the sample size was small, so it is not reasonable to extrapolate our data to the general population. In addition, the results of this study might have been influenced by the small sample size. Second, as we previously noted, we did not exclude patients with comorbidities such as hypertension and diabetes even if they were under treatment. It is possible that these comorbidities and their treatment affected the results. Third, as mentioned above, we did not have a planned control group without CPAP use. Therefore, we could not judge precisely whether changes in Ngal levels after CPAP implementation were caused by CPAP use or other reasons. Lastly, we did not measure C reactive protein (CRP) levels. Because inflammation has an influence on Ngal, results of measurement of high sensitive CRP would have been a good reflection of the inflammation status of patients and would have been helpful to achieve a more comprehensive understanding of the relationship between OSA and Ngal.

In summary, the present study provides the first clinical evidence demonstrating that plasma Ngal levels were positively but weakly associated with the severity of OSA. Plasma Ngal levels did not change after improvement in OSA, so we could not testify to the causality between Ngal levels and OSA severity. Because Ngal levels were just weakly correlated with the severity of OSA, changes in those levels appear to be influenced largely by other confounding factors. Thus, it would be difficult to use the Ngal level as a specific biomarker representing nocturnal hypoxia in OSA. The links between Ngal levels and metabolic syndrome remain controversial, and unrecognized determinants of plasma Ngal levels are likely to be present. Further studies are warranted to more comprehensively understand the regulation of Ngal in relation to OSA and metabolic syndrome.
